# Analysis of the antibacterial effects of turmeric on particular bacteria

**DOI:** 10.1097/MD.0000000000036492

**Published:** 2023-12-01

**Authors:** Edward Odogbu Odo, Josephine Adaku Ikwuegbu, Emmanuel Ifeanyi Obeagu, Silas Andrew Chibueze, Raphael E. Ochiaka

**Affiliations:** a School of General Studies (Physical and Health Education Unit) Michael Okpara University of Agriculture, Umudike, Nigeria; b Department of Medical Microbiology, College of Medicine & Health Sciences, Abia State University, Nigeria; c Department of Medical Laboratory Science, Kampala International University, Uganda; d Department of Biochemistry, Michael Okpara University of Agriculture, Umudike, Nigeria; e Department of Human kinetics and Health Education, Enugu State University of Science and Technology, Nigeria.

**Keywords:** bacteria, exercise, lipid profile, turmeric

## Abstract

Antimicrobial properties of plants have been investigated by a number of studies worldwide and many of them have been used as therapeutic alternatives because of their antimicrobial properties. The quest for suitable and affordable alternative in the face of increasing antimicrobial drug resistant has led researchers into exploring the use of plant extract in the treatment of infections. The antibacterial properties of turmeric (*Curcuma longa*) on selected bacteria were evaluated. Different concentrations of turmeric extract (100, 50, 25, and 12.5 mg/mL) were prepared using 2 solvents namely water and ethanol. The antibacterial activity was tested against *Bacillus* species, *Staphylococcus aureus, Escherichia coli* and *Pseudomonas aeruginosa* at different concentration of the extract using disc diffusion method and ciprofloxacin was the control. The zones of inhibition exhibited by ethanol and aqueous extracts against test organisms ranged from 1 to 10 mm. the ethanolic extracts were more effective than the aqueous extracts exhibiting zones of inhibition ranging from 3 to 10 mm against *Bacillus* species, 4 to 9 mm against *S aureus*, and 1 to 7 mm against *E coli*. There was no inhibitory effect against *P aeruginosa*. There was significant difference between the ethanol and aqueous extracts (*P* < .05). This study reveal that Turmeric plant has antibacterial potential against selected organisms and may be of great use of pharmaceutical industries for the development of medicine to cure ailments and control abnormal serum lipid profile.

## 1. Introduction

Components of turmeric are named curcuminoids, which include mainly curcumin (differuloyl methane), demethoxycurcumin, and bisdemethoxycurcumin.^[1]^ Curriculum is the most important fraction which is responsible for the biological activities of Turmeric.^[2]^ Since the time of Ayurveda (1900 CB), Turmeric (*Curcuma longa*) has been used for a wide variety of diseases and condition, including those of the skin, pulmonary, and gastrointestinal system, aches, pains, wound, sprains, and liver disorders. Extensive research on curcumin have demonstrated a wide spectrum of therapeutic effects such as anti-inflammatory, antibacterial, antiviral, anti-fungal, anti-diabetic, anticoagulant, hepatoprotective, anti-ulcer, hypotensive and hypocholesteremic.^[3,4]^ How a single agent could exhibit all these effects is an enigma under intense scrutiny.^[5]^ Curcumin possesses antibacterial property against a number of gram-positive and gram-negative bacteria.^[6]^ Also, its anti-inflammatory properties are well documented.^[4,7]^

## 2. Materials and methods

### 2.1. Study design

It was a cross-sectional study.

### 2.2. Sample size

200 g of Turmeric were used.

### 2.3. Ethical approval

Ethical approval was obtained from Research Ethics Committee of Abia State University, Abia State, Nigeria.

### 2.4. Collection of plant materials

The rhizomes of Tumeric (*C longa*) were purchased from Ahia Ohuru market Aba. The plant material was properly washed in running water to clean off the adhering sand particles, it was then rinsed afterwards. They were cut into pieces, air dried and made into powdered form using a clean blender.

### 2.5. Collection of test organisms

The test organisms such as *Staphylococcus aureus, Escherichia coli, Pseudomonas aeruginosa* and *Bacillus* species used in the study were obtained from Microbiology Laboratory of ABSUTH. All cultures were maintained on Nutrient agar slant at 4°C.

### 2.6. Extraction of plant materials

The extraction was carried out according to the method of Fatope et al^[8]^ Twenty grams (20 g) of powdered plant sample were mixed with 100 mL sterile distilled water and 100 mL 98% ethanol respectively. It was soaked for 24 hours at room temperature and filtered with Whatman No 1 filter paper and the filtrate was dried using hot air oven at 40°C. The extracts were labeled accordingly and stored into refrigerator for further analysis.

### 2.7. Sterility of extracts

0.1 g of each extract was added into 5 mL of sterile Nutrient broth. They were then incubated at 37°C for 24 hours. The extracts were clear after incubation indicating the absence of contaminant which could have caused a turbid appearance in the tube.

### 2.8. Preparation of sensitivity discs

Discs of 6 mm in diameter were punched out using Whatman No 1 filter paper with the aid of a paper punch and placed in Bijou bottles. The discs were then sterilized by autoclaving at 121°C for 15 minutes and allowed to cool. Cock solution of the Turmeric rhizomes ethanolic and aqueous extract (that were recovered) were prepared by dissolving 0.5 g (that is 500 mg) of each of the 2 extracts in 5 mL of dimethylsulfoxide. Each stock solution had a concentration of 100 mg/mL). From these stocks, 4 different concentrations of each of the extracts were prepared. These were 12.5, 25, 50, and 100 mg/mL. One hundred sterile discs were added into each concentration and allowed to absorb the solution. Since each paper disc absorbs 0.01 mL, the final disc concentration was 125, 0.25, 0.5, and 1.0 mg/mL respectively.

### 2.9. Preparation of McFarland solution

1 mL of concentrated sulfuric acid was added to 99 mL of distilled water to make 1% v v **solution sulfuric acid. Similarly, 0.5 g of dehydrated barium chloride (BaCl_2_, 2H_2_O) was dissolved in 50 mL of distilled water to make 1% w/v solution of BaCl_2_. Then 0.5 mL 1% BaCl_2_ was added to 99.5 mL 1% sulfuric acid solution and mixed properly.

### 2.10. Antibacterial susceptibility testing of the plant extracts

This was carried out using a procedure described by Bauer et al^[9]^ Using a sterile wire loop, 3 to 4 colonies of the test organism were emulsified in 3 mL of physiological saline. The turbidity was compared with McFarland turbidity standard 0.5 MFU (McFarland Unit). Using a sterile swab stick, the test organisms was inoculated onto sterile Iso-sensitest Agar plates. Prepared discs of the 4 different concentrations (that is 12.5, 25, 50, and 100 mg/mL) for each of the Turmeric extracts were placed on the inoculated plates. Ciprofloxacin was used as control. Plates were incubated aerobically at 37°C for 24 hours. The plates were examined for zones of inhibitions which were measured in millimeters (mm) using plastic ruler.

### 2.11. Statistical analysis

This was done using analysis of variance.

## 3. Results

Table 1 shows that ethanolic extract of Turmeric had zones of inhibition ranging 5-om 1 mm to 10 mm. The extract inhibited *S aureus*, and *Bacillus cereus* effectively at the concentration of 1.0, 0.5, and 0.25 mg/mL with zones of inhibition 9, 6, 4 mm respectively for *S aureus* and 10, 8, 3 mm respectively for *Bacillus* spp. The zones of inhibition increased as the concentration increased. The extract concentration of 0.25 mg/mL was unable show-zone of inhibition against *E coli* but the pathogen was inhibited at Q.5 mg/mL and 1.0 mg/mL with zones of inhibition 1 and 7 mm respectively. *Pseudomonas* species were not inhibited at all and also the extract concentration of 0.125 mg/mL had no effect on all the selected pathogens.

**Table 1:** Antibacterial activity of 98% ethanolic extract of turmeric on selection bacterial pathogens.

Zones of inhibition (mm)						
Concentration in mg/mL	*Staphylococcus aureus*	*Escherichia coli*	*Pseudomonas aeruginosa*	*Bacillus* spp	X^2^	*P* Value
1.0	9	7	0	10	12.44	.017
0.5	6	1	0	8		
0.25	4	0	0	3		
0.125	0	0	0	0		
Control	8	2	2	0		
(Ciprofloxacin)						

***Key:S**—**Staphylococcus.

E—Escherichia.

P—*Pseudomonas.*

Spp—Spcies.

Significant at *P* < .05.

Not significant at *P* > .05.

Aqueous extract of Turmeric had antibacterial activity on selected organisms except ion *Pseudomonas* with zones of inhibition ranging from 1 to 10 mm. The zones of inhibition increased as the concentration increased for *E coli* and *B cereus* but for *S aureus* the extract concentration of 0.5 mg/mL had no effect on the organism. Also, the extract concentration of 0.125 mg/mL had no effect on all selected organism (Table 2).

**Table 2:** Antibacterial activity of aqueous extract of turmeric on selection bacterial pathogens.

Zones of inhibition (mm)						
Concentration in mg/mL	*S aureus*	*Escherichia coli*	*Pseudomonas aeruginosa*	*Bacillus* spp	X^2^	*P* value
1.0	7	10	0	8	11.33	.022
0.5	0	5	0	5		
0.25	1	4	0	3		
0.125	0	0	0	0		
Control	5	2	1	0		
(Ciprofloxacin)						

**Key:S**—**Staphylococcus.

E—Escherichia.

P—*Pseudomonas.*

Spp—Species.

Significant at *P* < .05.

Not significant at *P* > .05.

## 4. Discussion

The result from our study shows that Turmeric extract had antibacterial activity. This is probably the reason some people use Turmeric for treatment of bacterial infections and also food industries use Turmeric as preservatives. The aqueous and ethanolic extract highly inhibited *S aureus, E coli, B cereus* at die concentration of 1.0 mg/mL, except *Pseudomonas* which showed no inhibition. The ethanolic extract gave a higher antibacterial activity on the test organisms than the aqueous extract. This is similar to the report of Sana and Ifra^[10]^ who reported that ethanolic extract of Turmeric shows a better result as compared to aqueous one. This may be due to better solubility of Turmeric in organic solvent resulting in the release of greater amount of active antimicrobial components.^[11]^ Using ethanolic extract, among different microorganisms *Bacillus specie* was found to be the most susceptible pathogen having a zone of inhibition ranging between 3 and 10 mm, followed by *S aureus* having a zone of inhibition ranging from 4 to 9 mm and *E coli* having a zone of inhibition ranging from 1 mm** -“mm. This finding agrees with the study of Onwuka^[12]^ who reported that *Bacillus subtilis* was the most sensitive organism to *C longa* extract of curcuminoid and oil. However, it contradicts with the finding of Parveen and Jehan^[13]^ who reported *S aureus* as the most susceptible pathogen.

The thick structural constituent of gram-positive organisms (*Bacillus* species and *S aureus*) *in* this study can be held responsible for the increase in the interaction between the active compound, curcumin and the structural lipoproteins. This increased interaction may result in the inhibition of gram-positive organisms.^[10]^ Wang et al^[14]^ reported that micro capsule curcumin was more effective against gram-positive organisms such as *S aureus.* Chandrana et al^[15]^ and Kim et al^[16]^ reported that Turmeric was effective against *E coli, B subtilis,* and *S aureus* which may be due to the presence of curcumimoid, a phenolic compound. Negi et al^[6]^ reported tumerone and curlone components of Turmeric possessed better antibacterial activity against a wide range of microbes including *B subtilis, S aureus, B cereus, Bacillus coagulans, E coli* and *P aeruginosa.* The antimicrobial of Turmeric is reported to be due to the presence of essential oil, curcumins, curcuminoids, Turmeric oil, turmeric and veleric acid.^[17,18]^ Odhav et al^[19]^ suggested that the mechanism of antibacterial action of spices involve hydrophobic and hydrogen bonding of phenolic compounds to membrane proteins, membrane disruption and destruction of electron transport system and cell wall disruption. The antimicrobial activity of aqueous extraction could be due to anionic components such i5 thiocyanate, nitrates, chlorides and sulfate in addition to many other compounds naturally present in plants.^[20]^

## 5. Conclusion

Our study reveals that Turmeric contains potential antibacterial components that may be of great use for the development of medicines by pharmaceutical industries as a therapy against abnormal serum lipid profile and various diseases. Turmeric extract possesses significant effect against test microorganisms. It is believed that with rising degree of antimicrobial resistance against the commonly and affordable antimicrobials, research should shift to the affordable alternatives which include the use of Turmeric for the treatment of bacterial diseases. The demonstration of antibacterial activity of Turmeric against bacteria in this study is an education that the plant *C longa* is a potential source for the production of antibacterial with a broad spectrum of activity. The result of this study also supports the traditional application of the plant and suggest that plant extract possesses compounds with antibacterial properties that can be used as antibacterial agent in novel drugs for the treatment of infections associated with the test organisms. We recommend that government, non-government organizations and philanthropists should encourage researchers in this field so that the spread of antimicrobial resistance would be curbed.

## Author contributions

**Conceptualization:** Edward Odogbu Odo.

**Data curation:** Edward Odogbu Odo.

**Investigation:** Edward Odogbu Odo.

**Methodology:** Edward Odogbu Odo, Emmanuel Ifeanyi Obeagu, Silas Andrew Chibueze.

**Supervision:** Edward Odogbu Odo.

**Validation:** Emmanuel Ifeanyi Obeagu.

**Visualization:** Edward Odogbu Odo, Emmanuel Ifeanyi Obeagu, Silas Andrew Chibueze, Raphael E. Ochiaka.

**Writing – original draft:** Edward Odogbu Odo, Josephine Adaku Ikwuegbu, Emmanuel Ifeanyi Obeagu, Silas Andrew Chibueze, Raphael E. Ochiaka.

**Writing – review & editing:** Edward Odogbu Odo, Josephine Adaku Ikwuegbu, Emmanuel Ifeanyi Obeagu, Silas Andrew Chibueze, Raphael E. Ochiaka.
